# Markov approach for inventory control with meta-heuristics in intermittent demand environment

**DOI:** 10.1371/journal.pone.0325658

**Published:** 2025-06-10

**Authors:** Ferhat Yuna, Burak Erkayman, Mustafa Yılmaz

**Affiliations:** 1 Department of Industrial Engineering, Faculty of Engineering, Ataturk University, Erzurum, Turkey; 2 Industrial Engineering and Business Information Systems, University of Twente, Enschede, The Netherlands; PLOS, UNITED KINGDOM OF GREAT BRITAIN AND NORTHERN IRELAND

## Abstract

Demand variability directly affects inventory management. The variability of intermittent demand causes high lost sales or holding costs. While lost sales reduce customer satisfaction, keeping excessive stock also creates high costs for companies. This situation can be prevented with an appropriate inventory policy. In this study, a Markov-based proactive inventory management approach supported by metaheuristic methods is proposed in the inventory management of intermittent demands. The main contribution of the proposed approach is to find a lower and upper limit for stock by modeling the intermittent demands in the past period with the Markov process. With these optimized limits, it is aimed to balance the largest costs caused by intermittent demands, namely stock and lost sales costs. The intermittent demands used were randomly generated in 4 different sizes from small to large. The proposed approach contributes to inventory management by minimizing the negativities caused by demand variability through the Markov process. A mathematical model has been proposed for stock level optimization, but no feasible solution has been found. The mathematical model was transformed into a fitness function and a solution was provided with the Tabu Search Algorithm and Simulated Annealing. The inventory management process of intermittent demand was first evaluated without the Markov approach, and then the Markov approach was included in the process. The results showed that the Markov approach was a good tool for inventory management of intermittent demand. When the results were examined, the stock limits computed with the Markov process balanced the increased inventory cost and lost sales costs due to intermittent demand.

## Introduction

When demand for a product is intermittent, tracking and managing product inventory is a difficult task. Concepts such as the reorder point, order quantity, or maximum stock level directly affect inventory management. These concepts are even more important for intermittent demands. Because intermittent demand contains zero demands. The occurrence of this different demand situation happens at random times. Therefore, this uncertainty causes high holding costs or high lost sales. Minimizing these costs is possible with a good demand forecast or inventory policy. The goal is to satisfy customer demand as best as possible. In doing so, it is necessary to minimize both holding costs and lost sales. The focus of this study is a min-max (s, S) inventory policy [1]. The place of this policy in this study is on finding a lower and upper inventory limit. However, the biggest difficulty here is that the demand is intermittent. This situation makes it significantly difficult to determine the optimum limits. To overcome this difficulty, a new policy is proposed that takes into account the lost sales specific to intermittent demand and reduces the negative effects of intermittent demand on costs.

It is necessary to distinguish between intermittent demand forecasts and intermittent demand inventory models. Many studies deal with point forecasting of intermittent demand, and the forecasting methods are compared in terms of the error of the forecasts. Intermittent demand inventory models are concerned with meeting customer demand rather than estimating the point. This is the point where this study was inspired. The objective of the study is to find an inventory model that increases the level of customer service by expressing it stochastically without predicting intermittent demand.

A two-state Markov chain was used by Willemain et al. [2] to express probabilistic intermittent demand. The first case is zero demand and the second case is non-zero demand. The transition probabilities of the corresponding demands were calculated from the historical data. This is the second focus of this study. That is, the first step is to estimate the sequence of zero and non-zero demands. This estimation is also performed with a first order Markov process. The second step is to estimate the size when the demand is non-zero. In this study, non-zero demands are assumed to derive from a probability distribution. Therefore, the first step is to determine the zero and non-zero demands. In the second step, the demand sizes for the non-zero demand are determined from the probability distribution. The use of the probability distribution in determining the size of demand means that future demand values can change probabilistically. Consequently, this Markov Process has enabled this study to take more account of real-life uncertainty.

The intermittent demands used by the proposed model and metaheuristic methods in this study are stochasticized by the Markov chain. The probabilities of the transition of the Markov chain are used to determine whether a demand exists in a period. This study is based on the assumption that the demand variable consists of any probability distribution if the demand for period i is non-zero. Thus, the new demand is generated according to the probability distribution in the periods in which the demand arises. Thus, the newly generated stochastic demand is used to determine the lower and upper stock limits in the proposed model. This is to prevent the proposed model from remembering the demand of the previous period and overfitting. The most important thing is to be able to make informed inventory decisions for the future. When demand is included in the model as probabilistic, the uncertainty of future periods is also included as probabilistic.

The main motivations of this paper are as follows:

1. Intermittent demand inventory problems are more important due to the difficult nature of intermittent demand.
2. Minimizing costs depends on good modeling of intermittent demand.
3. The Markov process is a good tool to explain intermittent demand processes.
4. Metaheuristic methods have been integrated with the Markov process and a good approximate solution has been produced.
5. Lost sales situation has been considered to create a more realistic perception.

The Markov chain-based approach has been proposed for the inventory management of intermittent demand with these motivations. This approach provides stock level optimization with metaheuristic methods. The main contribution of the proposed approach is the inclusion of the Markov process as an input to the inventory management of intermittent demands. This input is combined with metaheuristic methods to compute stock levels. The contribution of the Markov process in this study is to eliminate the negative effects of uncertainty on stock limits rather than estimating demand. In the first stage of the study, stock levels were computed with past period demands. In the second stage, the Markov process was included and it was observed that the computed stock levels were better. The results showed that incorporating the Markov process into intermittent demand inventory management has a positive effect.

The main contributions and unique aspects of this research are as follows:

1. Designing a metaheuristic-based the Markov process for inventory management of intermittent demands,
2. Designing a proactive inventory model based on probabilistic data for intermittent demands in future periods,
3. Proposing an inventory policy for future intermittent demands by simulating the intermittent demand of the past period with the Markov chain.

Based on this information, this study assumes that non-zero demands consist of a probability distribution. In this way, as mentioned, the intermittent demand is expressed as probabilistic with the Markov process. The mathematical model was modified to work with probabilistic demands. No feasible solution could be found with the proposed mathematical model. Then a fitness function is created. This fitness function is solved with TSA and SA and the results are recorded. The results were good because the demands were expressed stochastically.

## Literature

**Fig 1:**
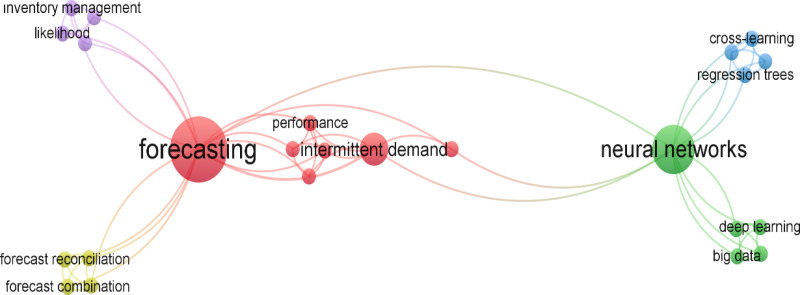
Network Analysis for the Intermittent Demand Keyword [3].

Much of the work done with intermittent demand consists of forecasting studies. This is clearly seen in the network analysis shown in Fig 1. Fig 1 was prepared using the Web of Science database with the VOSviewer package program.

The main time series-based methods proposed in the literature for intermittent demand forecasting for spare parts are listed in Table 1 [4]. In the table, the methods are classified according to their approaches. Although there are many forecasting methods, it is difficult to estimate the variability of intermittent demand and the zero-demand situation. This situation complicates the inventory management of intermittent demand products. A good forecast is helpful for good inventory management. However, the structure of intermittent demand does not allow this so easily. Therefore, a stochastic inventory model that incorporates uncertainty, rather than estimation, would be more beneficial. For this reason, after examining the estimation methods, the Markov process has been chosen for this study in order to meet the uncertainty of intermittent demands in future periods.

**Table 1:** Time Series Based Methods.

Category	Estimation Method
Traditional Time Series Approaches	Moving Average [5] Exponential Smoothing [6] Exponential Weighted Moving Average [7]
Modified Traditional Time Series Approaches	Adjusted Exponential Weighted Moving Average [8] Adjusted Holt Method [9] Holt-Winters Method [10]
Croston’s Method and Modified Versions	Croston’s Method [11] Syntetos and Boylan’s Method [12] Croston’s Modified Approach [13]
Bootstrap Method	Modified Bootstrap Method [14]
Demand Gathering/ Segregation	Filtering/ Clustering Method [15]

In modern times, studies on the automotive sector have intensified, especially due to the intermittent demand structure for spare parts. For example, Babai et al. [16] proposed a new estimation method using data sets from the automotive and military sectors. Again, Babai et al. [17] conducted an evaluation of the demand data they collected from the automotive industry and their forecasting methods. Pastore et al. [18] conducted a study of the bullwhip effect in an auto parts chain to explain how demand for different products varies. Petropoulos et al. [19] turned to intermittent demand forecasting and proved their forecasting performance using two spare parts demand data.

Dreyfuss and Giat [20] worked on an inventory system for the allocation of spare parts. Boutselis and McNaught [21] focused on predicting spare parts requirements for equipment failures, while Van der Auweraer and Boute [22] worked with critical spare parts for inventory management. For intermittent demand forecasting, Jiang et al. [23] proposed a support vector machine model with real data from the heavy vehicle industry, and a new method was developed by Li et al. [24] to estimate the demand for spare parts for war-damaged products. Panagiotidou [25] proposed a model to optimize spare parts ordering strategies. Tapia-Ubeda et al. [26] proposed an optimization model for inventory location optimization and inventory control decisions in the spare parts supply chain. Dombi et al. [27] proposed a new approach for forecasting by modeling the demand for electronic spare parts. Cavalieri et al. [28] have established decision mechanisms such as part classification, demand forecasting and stock management for maintenance spare parts within an enterprise.

Hua et al. [29] proposed a new approach that integrates the relationship between forecasting the occurrence of non-zero demands and non-zero spare parts demand. Kocer [30] proposed a modified Markov chain model (MMCM) for modeling and estimating intermittent demand data. Hasni et al. [31] used a Markov chain to link periods of demand (non-zero demand) and non-demand (zero demand). The data size and demand periods are treated separately. The Markov decision process was used by Lamghari-Idrissi et al. [32] for inventory decisions of spare parts, and a state- and time-dependent inventory policy was shown to be optimal and compared with heuristics. To estimate intermittent demand, a Markov-combined method (MCM) is proposed, which takes into account past sales and inventory status of the corresponding products [33].

The Wagner–Whitin [34] and Silver–Meal methods [35] work well when the demands are known. However, these methods cannot be used if the demands are unknown and lost sales are not allowed. However, lost sales may occur in the proposed model. This model focuses on a Markov-based approach to manage situations where the intermittent demands of future periods are unknown. An innovative inventory problem of using TSA and SA methods together with the Markov process to manage intermittent demand is discussed. To the best of our knowledge, this problem has not been studied in the literature for inventory management of intermittent demands using the Markov approach and metaheuristic methods.

Table 2 shows the categorization of intermittent demand studies using recent literature. The classification criteria include intermittent demand, demand simulation, inventory management, Markov process, stock level optimization, and metaheuristic methods.

**Table 2:** Reviewing related work.

Reference	Metaheuristic Methods	Stock Level Optimization	Intermittent Demand	Demand Simulation	Inventory Management	Markov Process
[34–35]	–	–	–	–	√	–
[30–31]	–	–	√	√	–	√
[32]	–	–	√	–	√	√
[33]	–	–	√	√	–	√
[16, 17, 18, 19, 27]	–	–	√	√	–	–
[21, 23, 24, 29]	–	–	√	√	–	–
[20,22]	–	–	√	–	√	–
[28]	–	–	√	√	√	–
[25–26]	–	–	√	–	√	–
This research	√	√	√	√	√	√

In particular, despite a comprehensive review of existing research, no stock level optimization study has been identified that integrates Markov process with metaheuristic methods for inventory management of intermittent demand. Therefore, this research presents a stock level optimization approach to intermittent demand inventory management by integrating Markov process and metaheuristics.

**Fig 2:**
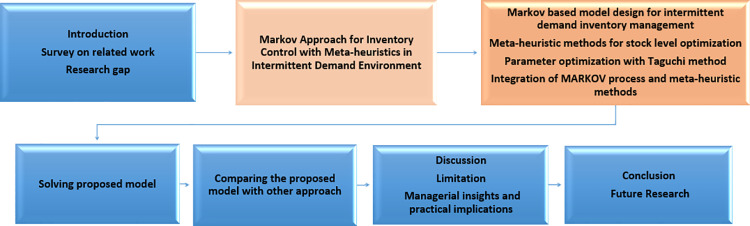
Research methodology in this research.

This topic involves formulating a Markov process that combines the constraints of intermittent demand, stock management, and stock level optimization. This formulation is supported by metaheuristic methods. Fig 2 shows the research methodology of the Markov-based approach proposed in this study. This research methodology outlines the study step by step and serves as a roadmap for the research process.

The proposed approach has significant benefits to managerial implications in intermittent demand inventory systems. The most important one is inventory level optimization. This optimization reduces the amount of lost sales and inventory costs. Order quantity and order time are determined by intermittent demand. Therefore, it also has a positive effect in terms of production planning. With correct stock levels, a buffer scenario is prepared against the negative effects of intermittent demand variability and zero demand situations.

## Methodology

### Problem definition

In the intermittent demand structure, periods when there is no demand or sudden increases in demand are observed. These situations make it difficult to maintain the correct inventory level. Keeping too much inventory increases inventory costs, while keeping less inventory creates lost sales, reducing customer satisfaction. Sudden increases in demand create problems in relations with suppliers due to the uncertainties in intermittent demand. The demanding nature of intermittent demand affects many business operations such as production, resource allocation, planning, supply chain and more.

In this study, stock level optimization is emphasized in order to reduce the effects of future demand uncertainty in inventory management of intermittent demand and to help overall operational efficiency. Stock levels are handled in two ways as upper and lower stock levels. However, while determining these levels, the Markov process was used in order to minimize the uncertainty about the future. The Markov process is designed to help determine future stock levels with the information it receives from past demands. In the proposed model, the levels found with the past period demands are compared with the stock levels computed by including the Markov process in terms of profit maximization.

**Fig 3:**
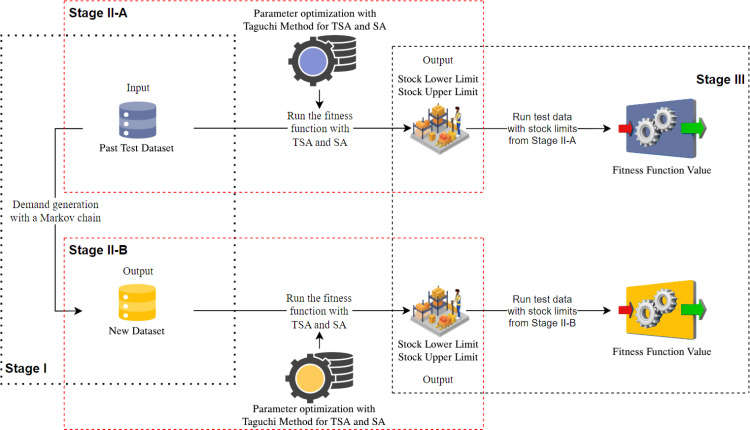
Flow of the inventory model with the proposed Markov approach.

The Markov-based intermittent demand inventory management approach proposed in Fig 3 has 3 stages. This approach aims to find the best stock levels (stock upper limit and stock lower limit) of an intermittent demand vector. The most important contribution of this approach is the inclusion of Markov process and metaheuristic methods in intermittent demand inventory management. In Stage I, the past intermittent demand data is simulated with a Markov chain. The purpose of this stage is to determine the behavior of the fitness function value in future demands. In Stage II-A, stock limits are computed with past period demands. In Stage II-B, stock limits are computed with new demands simulated with Markov. While making these calculations, parameter optimization was made with Taguchi method for both Stage II-A and Stage II-B. The fitness function value was computed by using the stock limits, which are the outputs of Stage II, in a test data. According to these fitness function values, it has been observed that in Stage III, the results from the Markov approach are better than the results from the past period.

### Markov process

The Markov process is a technique of operations research. It is a technique that makes it possible to obtain information about the future status of an activity that is taking place now [36]. It is called a stochastic process if the states in any system under study can change with a certain probability at constant or random times. Determining their future states by using the probabilities of the states in the system forms the basis of the Markov process. Markov analysis is used in situations where there is a stochastic transition from one state to another in the system. Here, the relevant transitions are expressed in terms of transition probabilities [37].

### Markov property

For a random process to be a Markov chain, it must have the following basic properties [38]:

If $P=\{\;X_{t+1}=j|X_{0}=k_{0}{,X}_{1}=k_{1},\ldots \ldots ,X_{t-1}=k_{t-1},X_{t}=i\}=P\{\;X_{t+1}=j\;|X_{t}=i\}$ for t=(0,1,2,3...), the random process $X_{t}\;$has the Markov property. In other words, each state of the process depends on the previous state. This independence property is called the Markov property.

The Markov process is called a chain under certain conditions:

- It has the Markov property.
- The possible states are a finite set.
- The initial probabilities are known.
- There are stationary transition probabilities.

Below is a transition probability matrix. The matrix $P_{ij}$ gives the probability that the process that is in state i in period t will be in state j in period t + 1, i.e., the transition probability.


$$P_{ij}=\;[\begin{array}{ccc} P_{11}\;P_{12} & \cdots & P_{1S}\\ P_{21}\;P_{22} & \cdots & P_{2S}\\ \vdots & \ddots & \vdots \\ P_{S1}\;P_{S2} & \cdots & P_{SS} \end{array}]$$

Since $P_{ij}\;$ contains transition probabilities, this matrix is called a stochastic matrix. In the matrix, $\sum_{j}\;P_{ij}$=1 for each i value and $P_{ij}\geq 0$ for each i and j. When these conditions are satisfied, the Markov chain is defined [39].

If the random variable X(t) can be observed as a function of time t, then X(t) is a continuous-time stochastic process. If the random variable X is observed at specific times in time t, then X(t) is a discrete-time stochastic process. The random variable X can also be discrete or continuous. The problem in this study fits into the class of discrete state and discrete time stochastic processes. Cases are determined as S={0,1} (0: there is no demand in period i 1: there is demand in period i). In one-period time intervals, it is checked whether there is a situation with zero demand.

### Illustrative example

In this section, the 1st test data (250 demands) from 4 test data is shown as an example. The transition probabilities and probability distributions of other test data are given in Table 7 in the Fitness Function and Test Data section. In order to classify the demand as intermittent, some statistical information is needed. ADI (Average Demand Interval) calculates the average interval between two consecutive demands. ${CV}^{2}$ (Coefficient of Variance) expresses the variation of the standard deviation with respect to the mean. The ADI value of the generated demand is 1.3661 and the ${CV}^{2}$ value is 0.0893. Randomly generated demand belongs to the class of intermittent demand with these values.

**Table 3:** Transition probability matrix of 250-period demand.

$P_{ij}$		Zero (0)	Non-zero (1)
**Zero (0)**	0.2727	0.7273
**Non-zero (1)**	0.2678	0.7322

**Table 4:** Statistical information on non-zero demands.

Distribution Summary	Chi Square Test
Distribution:Normal Expression: NORM (30.8, 9.17) Square Error:0.004200	Number of intervals = 13 Degrees of freedom = 10 Test Statistic = 5.13 Corresponding p-value> 0.75
**Data Summary**	**Histogram Summary**
Number of Data Points = 183 Min Data Value = 5 Max Data Value = 54 Sample Mean = 30.8 Sample Std. Dev. = 9.2	Histogram Range = 4.5 to 54.5 Number of Intervals = 50

**Table 5:** Notation List.

Indices	
T={$T_{1},T_{2},T_{3},T_{4}$}	Number of intermittent demand periods
**Parameters**	
$C_{P}$	Product cost (Unit: Dollar Value:10)
$C_{o}$	Order cost (Unit: Dollar Value:10)
$C_{h}$	Holding cost (Unit: Dollar Value:0.2)
$C_{LS}$	Lost sale cost (Unit: Dollar Value:0.2)
P	Sale price (Unit: Dollar Value:100)
$D_{i}$	Amount of demand in period i (Unit: Number)
**Variables**	
$I_{LB}$	Lower stock limit
$I_{UB}$	Upper stock limit
$y_{i}$	1 if order is opened in period i, otherwise 0
$u_{i}$	1 if there is lost sale in period i, otherwise 0
${k1}_{i}$,${k2}_{i}$	Variable related to order
${k3}_{i}$,${k4}_{i\;}$	Variable related to lost sales
$Q_{i}$	Amount of order in period i
$I_{i}$	Amount of stock in period i
${LS}_{i}$	Amount of lost sales in period i
$S_{i}$	Amount of sales in period i

**Table 6:** Probability distributions of the test data and transition probabilities.

TN	NP	ADI	${CV}^{2}$	Probability Distribution Parameters		
1	250	1,3661	0,0893	NORM(30.8, 9.17)		
				**Transition Probabilities**		
					**0**	**1**
				**0**	0,2727	0,7273
				**1**	0,2678	0,7322
2	10000	1,4665	0,0394	NORM(49.9, 9.91)		
				**Transition Probabilities**		
					**0**	**1**
				**0**	0,3085	0,6915
				**1**	0,3226	0,6774
3	250000	1,5404	0,0312	NORM(48.3, 8.52)		
				**Transition Probabilities**		
					**0**	**1**
				**0**	0,3506	0,6494
				**1**	0,3509	0,6491
4	500000	1,5645	0,0293	NORM(47.5, 8.13)		
				**Transition Probabilities**		
					**0**	**1**
				**0**	0,3593	0,6407
				**1**	0,3617	0,6383

***TN:*** *Test data number* ***NP:*** *Number of periods.*

**Table 7:** Taguchi experimental design and experimental data for SA.

		Parameters of the SA				Fitness Function Value		
		Annealing Function	Reannealing Interval	Temperature Update Function	Initial Temp.	First Run	Second Run	Third Run
**Experiment**	1	Fast Annealing	150	Exponential	250	5515226,8	5515084,8	5515377,9
	2		150	Logarithmic	500	5514622,8	5513662,6	5515062,4
	3		150	Linear	750	5515212,4	5515225,9	5513250,4
	4		300	Exponential	250	5514865,8	5515100,2	5515302,8
	5		300	Logarithmic	500	5513766,5	5514432,3	5514653,8
	6		300	Linear	750	5515251,3	5506226,0	5515254,7
	7		500	Exponential	500	5515213,6	5515278,8	5515390,6
	8		500	Logarithmic	750	5515124,4	5514972,2	5515270,2
	9		500	Linear	250	5515275,1	5515218,3	5514865,1
	10	Boltzman Annealing	150	Exponential	750	5515390,6	5515278,8	5515302,7
	11		150	Logarithmic	250	5515179,6	5515215,7	5514770,6
	12		150	Linear	500	5515094,2	5515301,5	5515215,6
	13		300	Exponential	500	5515226,8	5515226,8	5514865,7
	14		300	Logarithmic	750	5515092,9	5515209,2	5514849,5
	15		300	Linear	250	5515291,3	5514175,7	5515204,3
	16		500	Exponential	750	5515390,5	5515213,6	5515277,0
	17		500	Logarithmic	250	5515075,0	5515146,9	5515250,3
	18		500	Linear	500	5515212,2	5515286,5	5514821,1

**Fig 4:**
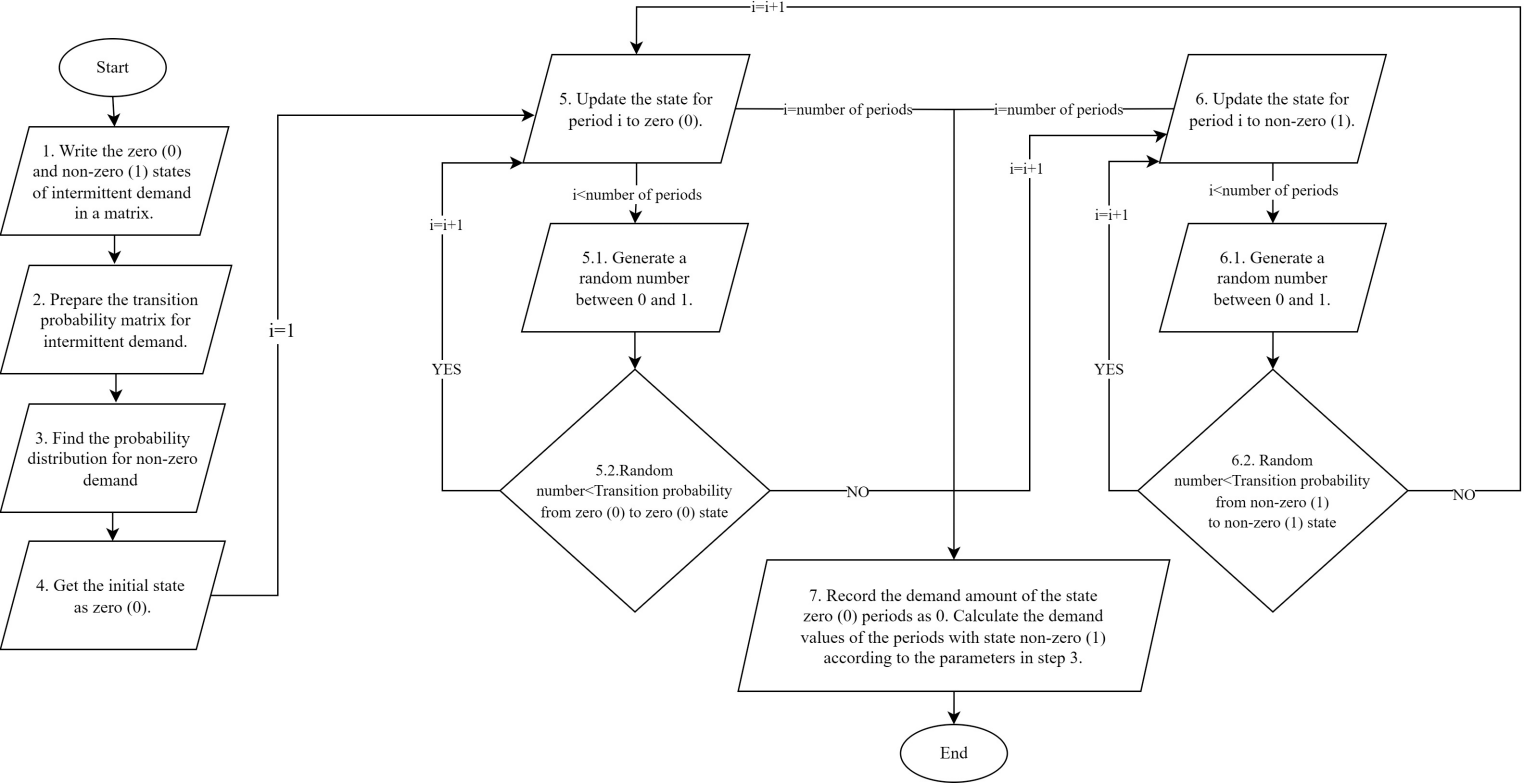
Flowchart of intermittent demand generation with Markov chain.

In the proposed procedure, the first step is to determine whether there is a demand for period i. If there is a demand, a new demand is generated according to the probability distribution of the demand. Whether the demand occurs or not is determined by the Markov chain. As mentioned above, the relevant process is a stochastic process with discrete state and discrete time. The states are denoted by S={0,1} (0: there is no demand in period i 1: there is demand in period i). How to create a stochastic intermittent demand is explained step by step in Fig 4.

As shown in the first step in Fig 4, intermittent demands are classified into zero (0) and non-zero (1) states. For these two cases, the transition probability matrix of intermittent demand is prepared. Probability distribution is calculated for intermittent demands belonging to the non-zero (1) class. While deriving the demands to be operated for stock level optimization, the initial state is determined as zero (0) in step 4. Markov chain is used to determine the state of the next period. A random number between 0 and 1 is derived. If the generated random number is smaller than the probability of the current state returning to itself, the next state will still be the same. However, if the random number is greater than the probability of the current state returning to itself, the transition to the other state is made in the next period. These processing steps continue until the size of the intermittent demand under consideration is reached. After all states are determined, the demand for non-zero (1) states is derived according to the probability distribution in step 3 in Fig 4.

The calculated transition probabilities are shown in Table 3. According to this table, the probability of transition from state 0 to state 0 is 0.2727, the probability of transition from state 0 to state 1 is 0.7273, the probability of transition from state 1 to state 0 is 0.2678, and the probability of transition from state 1 to state 1 is 0.7322.

**Fig 5:**
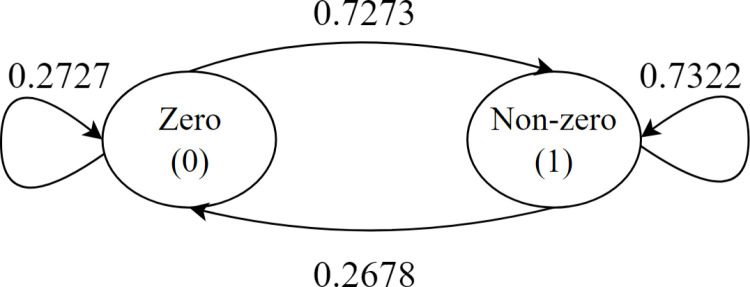
Diagram of transition probabilities for 250 demands.

The transition probabilities given in Table 3 are shown as a diagram of transition probabilities in Fig 5. For example, the value 0.2727 was obtained by dividing the number of transitions from state 0 to state 0 by the number of transitions from state 0 to states 0 and 1. As you can see, it satisfies the conditions $\sum_{j}\;P_{ij}$=1 for each i value in the Markov property and $P_{ij}\geq 0$ for each i and j.

Non-zero demands were selected and the probability distribution was determined in the Arena Input Analyzer program.

**Fig 6:**
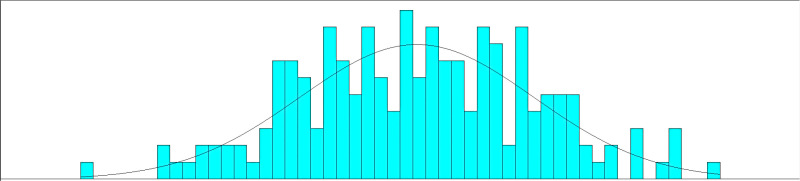
Histogram of non-zero demands.

The histogram of the demand is shown in Fig 6. The details of the information of this histogram can be found in Table 4.

In the proposed procedure, the zero or non-zero demand states for period i are first determined. If non-zero demand is present, demand is generated according to the corresponding probability distribution. Thus, the sizes of non-zero demand are determined. Assuming that there is no demand in period t = 0, the stochastic demand is formed according to the transition probabilities in Table 2 and the NORM (30.8, 9.17) distribution found in the input analysis.

### Mathematical model

Table 5 gives the notation list where the indices, parameters and decision variables of the mathematical model are defined.

The proposed mathematical model aims to find the values of $I_{UB}\;$and $I_{LB}$. These values are the stock lower and upper limit values. Profit maximization is aimed with the stock limit values found.


$$\begin{array}{c} Max\;\sum_{i=1}^{T}\;(\;P*S_{i}-C_{p}*Q_{i}-C_{LS}*{LS}_{i}-C_{h}*I_{i}-C_{o}*y_{i}) \end{array}$$ (1)

Subject to:


$$Q_{0}=0$$ (2)

$$I_{0}=0$$ (3)

$$\begin{array}{c} I_{i}=I_{i-1}+Q_{i-1}-D_{i}\;{\;\forall }_{i}\in \;T \end{array}$$ (4)

$$\begin{array}{c} {k1}_{i}-{k2}_{i}=I_{LB}-I_{i}\;{\;\forall }_{i}\in \;T \end{array}$$ (5)

$$\begin{array}{c} {k1}_{i}\leq M*\;y_{i}\;{\;\forall }_{i}\in \;T \end{array}$$ (6)

$$\begin{array}{c} {k2}_{i}\leq M*\left(1-y_{i}\right){\;\forall }_{i}\in \;T \end{array}$$ (7)

$$\begin{array}{c} Q_{i}\geq \left(I_{UB}-I_{i}\right)*y_{i}\;{\;\forall }_{i}\in \;T \end{array}$$ (8)

$$\begin{array}{c} {k3}_{i}-{k4}_{i}=D_{i}-I_{i}\;{\;\forall }_{i}\in \;T \end{array}$$ (9)

$$\begin{array}{c} {k3}_{i}\leq M*\;u_{i}\;{\;\forall }_{i}\in \;T \end{array}$$ (10)

$$\begin{array}{c} {k4}_{i}\leq M\left(1-u_{i}\right){\;\forall }_{i}\in \;T \end{array}$$ (11)

$$\begin{array}{c} {LS}_{i}\geq \left(D_{i}-I_{i}\right)*u_{i}\;{\;\forall }_{i}\in \;T \end{array}$$ (12)

$$\begin{array}{c} S_{i}=D_{i}-{LS}_{i}\;{\;\forall }_{i}\in \;T \end{array}$$ (13)

$$I_{LB}{\leq I}_{UB}$$ (14)

$$\begin{array}{c} {k1}_{i},{k2}_{i},{k3}_{i},{k4}_{i\;},Q_{i},I_{i},{LS}_{i},S_{i}\geq 0\;{\;\forall }_{i}\in \;T \end{array}$$ (15)

$$I_{UB},I_{LB}\geq 0$$ (16)

$$\begin{array}{c} y_{i},u_{i}\in \left\{0,1\right\}\;{\;\forall }_{i}\in \;T \end{array}$$ (17)

The objective function (1) is computed as the difference between revenue from product sales and lost sales, holding, ordering and product costs. Equation (2): The initial order quantity is zero. Equation (3): The initial stock quantity is zero. Equation (4): The stock of period *i* is updated according to the stock in period (*i-1*), orders in period (*i-1*) and demands in period *i*. Equations (5), (6), (7) and (8): This constraint group controls the orders and operates according to the Min-max (s,S) policy. If the stock quantity in period *i* is less than $I_{LB}$, an order is placed, otherwise no order. Equations (9), (10), (11) and (12): This constraint group controls lost sales. If the stock quantity in period *i* is less than the demand in period *i*, lost sales will occur, otherwise no lost sales will occur. Equation (13): Controls the quantity of products sold. If lost sales are zero, the quantity of product sold is equal to demand. Equation (14): Controls stock limits. The lower stock limit cannot exceed the upper stock limit. Equations (15), (16) and (17): Defines the type of decision variables. The mathematical model was solved by Yuna et al. [40] and no feasible solution was found. Therefore, meta-heuristic methods were used in this study. This work has been modified as shown in Fig 3 and Fig 9. Thus, demand uncertainty in future periods affected the model less.

**Fig 7:**
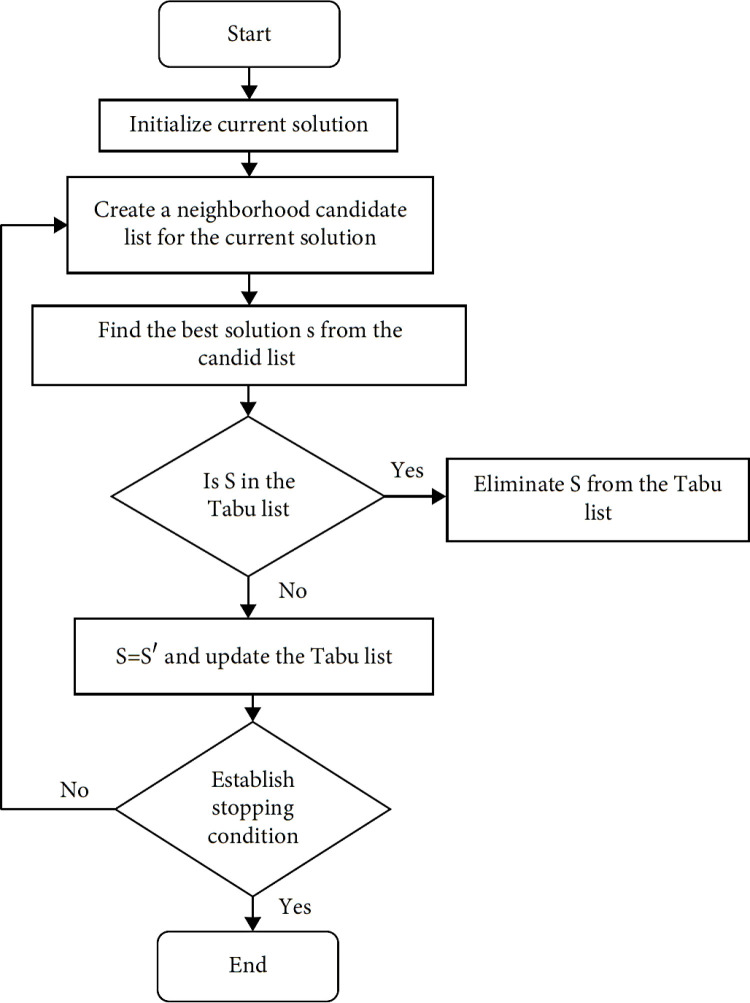
Flowchart for the Tabu Search Algorithm [43].

**Fig 8:**
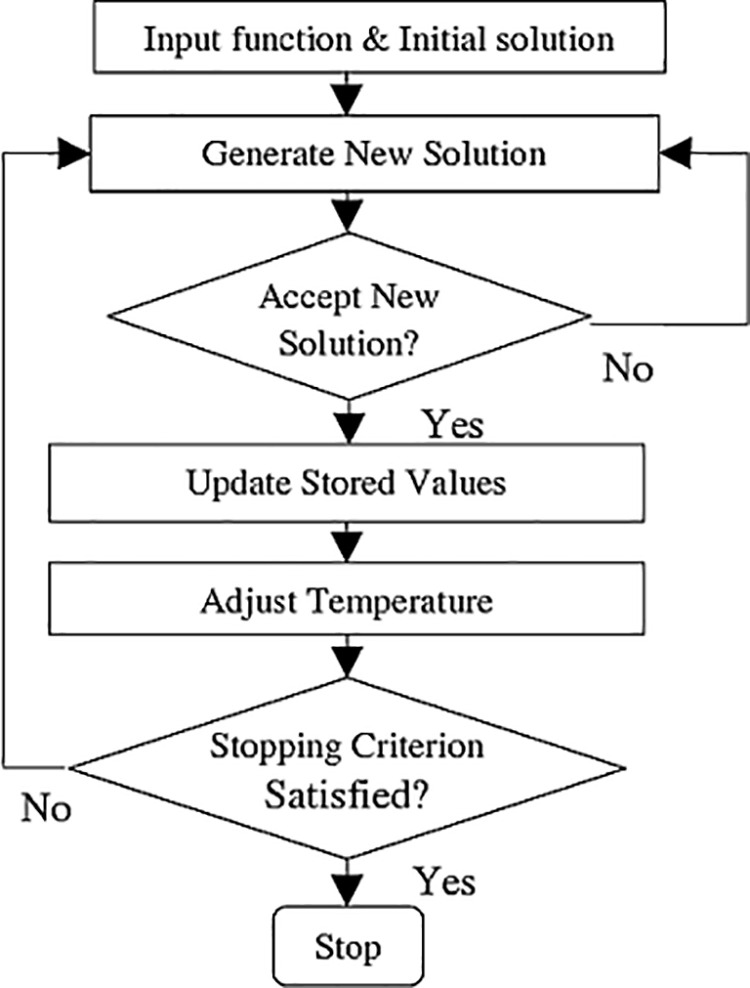
Flowchart for the Simulated Annealing [45].

### Tabu search algorithm (TSA) and simulated annealing (SA)

TSA was first proposed by Fred W. Glover in 1986 and formulated in 1989 [41]. Glover designed the TSA to avoid the local optimum problem. It has been proposed for solving combinatorial problems. TSA has been successfully applied in many problems such as personnel planning, character recognition, architectural design, telecommunication routing, quadratic assignment problems, traveling salesman problem, graph painting [42]. As you can see, TSA has a very wide usage area. Therefore, TSA was preferred for the problem proposed in this study. The method tries to reach better solutions iteratively, starting from an initial solution. The TSA prohibits certain actions in order to seek solutions in new areas and to avoid repetitive solutions. These movements are stored in a temporary memory and recorded in the tabu list, taking the name tabu. The Tabu List has a short-term memory. When the memory period is over, the relevant movements are removed from the tabu list and can be re-evaluated. Although it is tabu, there may be solutions to be evaluated. In this case, the tabu breaking criterion must be met. The flow chart of TSA is given in Fig 7.

SA is a random search method proposed by Kirkpatrick et al. [44] to solve optimization problems. SA gets its name from the method of physical annealing of solids. It has been applied to large-scale problems and has achieved successful results. SA avoids the local optimum problem like TSA and is a widely used method with its convergence feature. Therefore, another metaheuristic chosen for the proposed problem is SA. SA does not always focus on good solutions when improving iteratively. With a certain probability it also accepts bad solutions. Thus, it avoids the local optimum. The flow chart of SA is given in Fig 8.

Intermittent demand problems are more suitable for solving with metaheuristic methods due to the uncertainty in demand and the discontinuous structure. The main reason for preferring TSA and SA in this study is the success of these methods in practice. TSA and SA methods have shown successful results in many inventory management problems in the industry and are algorithms that have proven to be applicable in practice [46]. In fact, the approach proposed in this study is an inventory management approach. Therefore, it is important to select algorithms that are successful in the field of inventory management. Another reason is that TSA and SA have a flexible structure that can be adapted to different problem structures. Thanks to this flexibility, the uncertain structure of intermittent demand can be modeled quite well with these methods.

For TSA, the effects of tabu list length and number of iterations on the fitness function value were investigated. For SA, the effects of annealing function, reannealing interval, temperature update function and initial temperature parameters were investigated. The Taguchi method was used to determine the most appropriate values of the relevant parameters.

### Fitness function and test data

**Fig 9:**
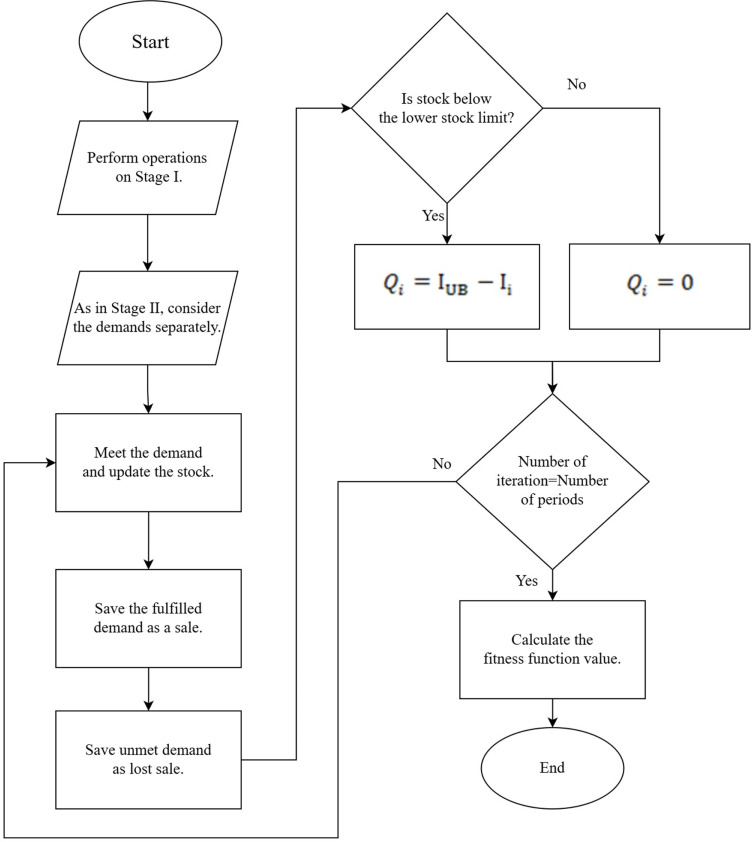
Flowchart of proposed fitness function algorithm.

A fitness function is proposed that fully satisfies the proposed mathematical model. The value of the fitness function expresses the value of the objective function of the mathematical model. No feasible solution could be found with the mathematical model. Therefore, a solution was sought to the problem with fitness function and meta-heuristic methods. The fitness function computes equation 1 in the mathematical model. All processes in the mathematical model such as orders, stocks, lost sales, and sales are the same in this function. The proposed function only needs a stock lower and upper limit as input. This input is optimized with meta-heuristic methods. The solution was sought using the tabu search algorithm and simulated annealing. The flow chart of the fitness function created for these algorithms is shown in Fig 9.

Table 6 shows 4 test data, the transition probabilities of these test data and the probability distributions of demands in the non-zero demand parts. These data, which belong to the intermittent demand class, contain a demand of size 1x (number of periods). As indicated in Table 6, non-zero demands are normally distributed. In fact, one of the main assumptions of this study is that non-zero demands express a probabilistic distribution. As mentioned in the flowchart of intermittent demand generation with the Markov chain, the transition probabilities of the test data were calculated. These probabilities and normal distribution parameters are used in generating the stochastic demand from the test data.

The limits that must be met in ADI and ${CV}^{2}$ statistics for the test data given in Table 6 to be classified as intermittent demand are mentioned in Illustrative Example section. The ADI value of the intermittent demand must be greater than 1.32 and the

${CV}^{2}\;$value must be less than 0.49. These limits were computed in the study of Syntetos which is the main reference for many intermittent demand studies [47]. All of the 4 different sized intermittent demands derived for this study satisfy the conditions for intermittent demand (ADI > 1.32 and ${CV}^{2}$<0.49). The transition probability matrix and the probability distributions of the demands to be used with the proposed Markov approach are also shown in Table 6. These 4 data were run separately for the proposed approach and the results are shown in Results and Discussion section.

The main assumption of the study is that past period demands are known and are intermittent demands. In order to support this assumption, ADI and ${CV}^{2}\;$values of 4 test data were calculated. The calculated statistics were interpreted based on the limits determined by the main reference Syntetos [47]. It is seen that 4 data created according to these limits fall into the intermittent demand class. Another assumption is the assumption that non-zero demands in intermittent demand come from a probability distribution. This assumption is also shown in Table 6 that after providing ADI and ${CV}^{2}$ statistics for intermittent demand, non-zero demands conform to a normal distribution.

In order to clarify the solution procedure, the proposed approach is briefly summarized as follows:

1. A new data set was generated from 4 test data using the Markov process as shown in Fig 4. This stage constitutes Stage I.
2. In Stage II, both past test data and new data generated by the Markov process were used. For both data, TSA and SA were run with the fitness function shown in Fig 9. In addition, parameter optimization was performed with the Taguchi method during this run.
3. Both data sets were run separately using the optimized parameters in TSA and SA.
4. In these processes, Stage II-A is the stage where past data is used, and Stage II-B is the stage where data generated from the Markov process is used.
5. Stock levels were computed for each data set as a result of Stage II-A and Stage II-B. Stock levels from these two stages were run with test data in Stage III. Thus, fitness function values belonging to Stage III were computed.

Finally, the results are shown comparatively in Table 12 and Fig 12.

**Table 8:** Taguchi experimental design and experimental data for TSA.

	Parameters of the TSA		Fitness Function Value		
Experiment	Tabu List Length	Number of Iterations	First Run	Second Run	Third Run
1	50	50	5513125,7	5514549,6	5514251,2
2	50	100	5514504,4	5513072,1	5513611,6
3	50	150	5514853,4	5514045,1	5515099,3
4	50	200	5514771,2	5514614,4	5514402,1
5	100	50	5514435,5	5513568,4	5514267,0
6	100	100	5514709,9	5513472,5	5514928,3
7	100	150	5514138,4	5512722,1	5513847,9
8	100	200	5514917,3	5514328,2	5513544,5
9	150	50	5513147,4	5512209,6	5513976,1
10	150	100	5514847,3	5513965,1	5514588,4
11	150	150	5513568,9	5514634,4	5514710,3
12	150	200	5514871,8	5514795,7	5514882,9
13	200	50	5514182,2	5514159,9	5513827,7
14	200	100	5514574,0	5514062,4	5513949,4
15	200	150	5514872,4	5514373,6	5513503,3
16	200	200	5514199,6	5514668,1	5514461,1

**Table 9:** The parameters determined as a result of the experimental designs.

Parameters of the SA				Parameters of the TSA	
Annealing Function	Reannealing Interval	Temperature Update Function	Initial Temperature	Tabu List Length	Number of Iterations
Boltzman Annealing	500	Exponential	250	200	200

**Table 10:** TSA Solutions.

*Tabu Search Algorithm Solutions*							
*TN*	*NP*	*Past Data with TSA*			*Markov Process with TSA*		
		*SLL*	*SUL*	*FFVTD*	*SLL*	*SUL*	*FFVTD*
1	250	87	108	**5.4730e + 06**	103	167	**5.4731e + 06**
2	10000	149	178	3.3443e + 08	147	175	3.3445e + 08
3	250000	126	163	**7.7503e + 09**	153	165	7.7527e + 09
4	500000	157	160	**1.4995e + 10**	143	392	1.4995e + 10

***TN*** *Test data number* ***NP:*** *Number of periods* ***SLL****: Stock lower limit,* ***SUL****: Stock upper limit,* ***FFVTD****: Fitness function values of the test data.*

**Table 11:** SA Solutions.

*Simulated Annealing Solutions*							
*TN*	*NP*	*Past Data with SA*			*Markov Process with SA*		
		*SLL*	*SUL*	*FFVTD*	*SLL*	*SUL*	*FFVTD*
1	250	89	180	5.4727e + 06	97	136	5.4727e + 06
2	10000	143	217	**3.3444e + 08**	143	168	**3.3446e + 08**
3	250000	127	128	7.7201e + 09	151	156	**7.7529e + 09**
4	500000	134	191	1.4994e + 10	143	152	**1.4995e + 10**

***TN*** *Test data number* ***NP:*** *Number of periods* ***SLL****: Stock lower limit,* ***SUL****: Stock upper limit,* ***FFVTD****: Fitness function values of the test data.*

**Table 12:** Differences between FFVTD at Stage II-A and Stage II-B.

*TN*	*NP*	Stage II-B	Stage II-A	*Difference in FFVTD* *Between Two Approaches*
1	250	5,4731E + 06	5,4730E + 06	1,0000E + 02
2	10000	3,3446E + 08	3,3444E + 08	2,0000E + 04
3	250000	7,7529E + 09	7,7503E + 09	2,6000E + 06
4	500000	1,4995E + 10	1,4995E + 10	0,0000E + 00

***TN:*** *Test data number* ***NP:*** *Number of periods* ***FFVTD****: Fitness function values of the test data.*

**Fig 10:**
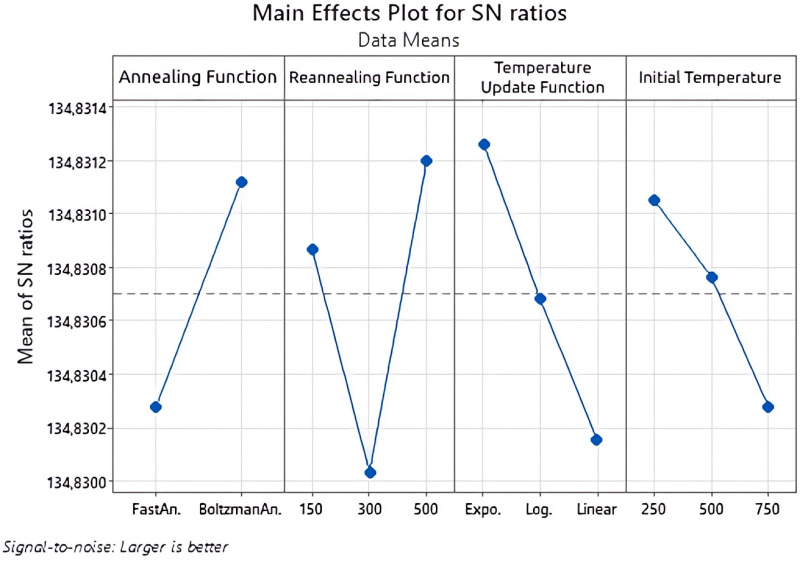
Effects of the factors for SA.

**Fig 11:**
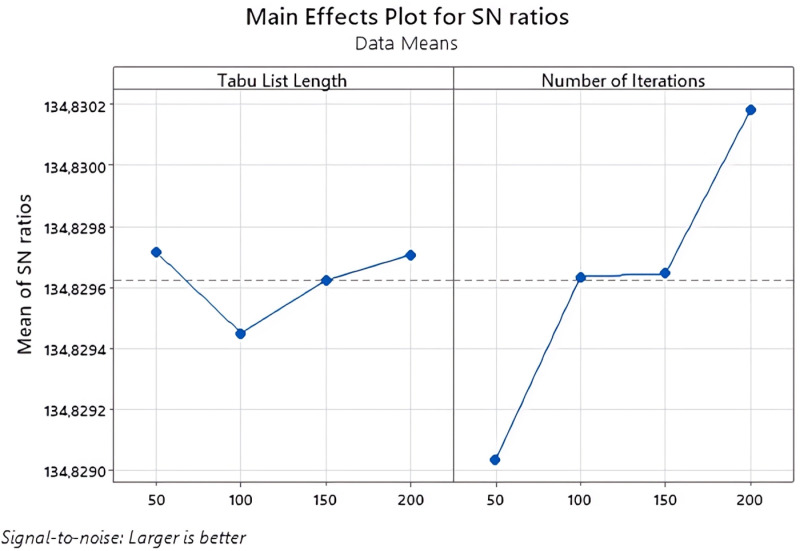
Effects of the factors for TSA.

**Fig 12:**
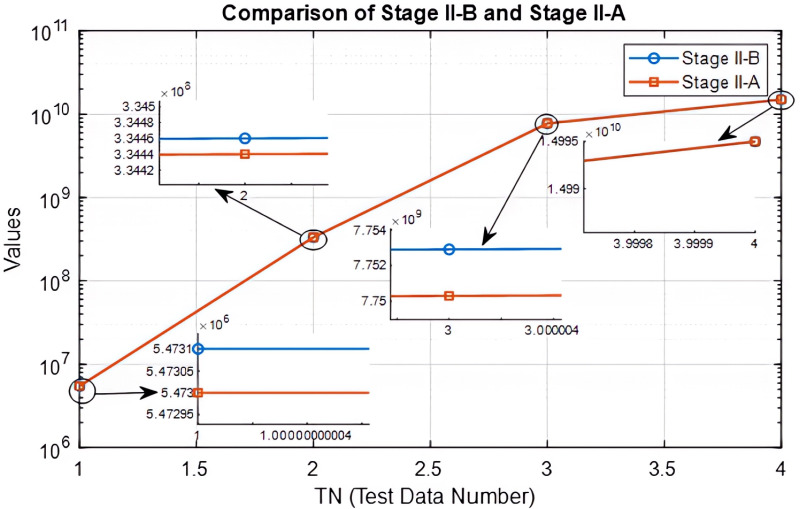
Comparison of Stage II-B and Stage II-A.

## Results and discussion

Focusing on the use of the proposed approach with TSA and SA methods and the ability to produce better results, a need for parameter optimization in these methods has arisen. In this section, the Taguchi method for parameter optimization, the fitness function values computed with the optimized parameters of TSA and SA, and a comparative analysis of the proposed Markov process approach are presented.

### Taguchi method

The Taguchi method is an effective method that evaluates how an event is affected by factors at different levels [48]. This method aims to give the results of the full factorial study with a small number of experiments. With the Taguchi method, it is possible to reach the results in a short time with less experiments in optimization studies. Experimental designs are used to determine the optimum values of controllable parameters [49,50].

In this study, the Taguchi method was used to determine the appropriate parameters while calculating the value of the proposed fitness function with TSA and SA. The experimental design for SA is shown in Table 8 and the experimental design for TSA is shown in Table 9. The larger-best approach was used while determining the most appropriate parameters according to the results of the experimental designs. Because the focus of the study is profit maximization. The signal-to-noise ratio measures how the response changes relative to the target value under different noise conditions. Depending on the purpose of this study, the biggest-best approach among the signal-to-noise ratios was adopted.

As seen in Table 7, the L_18_(2^1^3^3^) orthogonal experiment design plan was used to determine the most appropriate parameters of SA. The Taguchi experiment results of the fitness function obtained as a result of the L_18_(2^1^3^3^) orthogonal experiment design plan used in the study and three different repetitions operated under the same conditions are given in Table 7.

Performance criteria for the computed fitness function values are shown on the y-axis and parameter levels are shown on the x-axis. The effects of factors are given for SA in Fig 10 and for TSA in Fig 11. The point where the performance criteria have the highest value indicates the level at which the relevant parameter is the best. As seen in Fig 10, the best level for the Annealing Function was Boltzman Annealing, the best level for the Reannealing Interval was 500, the best level for the Temperature Update Function was Exponential and the best level was 250 for the Initial Temperature.

As seen in Table 8, the L_16_(4^2^) orthogonal experiment design plan was used to determine the most appropriate parameters of TSA. The Taguchi experiment results of the fitness function obtained as a result of the L_16_(4^2^) orthogonal experiment design plan used in the study and three different repetitions operated under the same conditions are given in Table 8.

In the experimental design for TSA, the point where the performance criteria have the highest value indicates the level at which the relevant parameter is the best. As seen in Fig 11, the best level for Tabu List Length was found to be 200 and the best level for Number of Iteration was 200.

### Numerical results of proposed model

For SA and TSA, the parameter levels used in the runs after this stage are given in Table 9. These parameters were run first with the past data and then with the data produced by the Markov Chain.

The upper and lower stock limits taken from both data were used in the fitness function calculation of the test data. Along with the proposed fitness function, the tabu search algorithm and simulated annealing were first run on the past test data and the results were recorded. Subsequently, stochastic data of the same dimensions were generated from the test data using the Markov chain. TSA and SA were run on the stochastic data and the results were recorded. In Tables 10 and 11, stock limits and results for both TSA and SA are given. These results are shown separately for both past data and Markov process.

According to Table 10, stock limits computed with the help of TSA and fitness function values of these limits are shown.

According to Table 11, stock limits computed with the help of SA and fitness function values of these limits are shown. The bolded results are determined as follows: For example, for TN = 1, the FFVTDs computed with Markov are 5.4731e + 06 for TSA and 5.4727e + 06 for SA. The larger of these values is selected as 5.4731e + 06 for TSA and is indicated in bold.

### Discussion and comparative results of the proposed model

As mentioned in Methodology section, Stage II-A is the stage where the Markov process is not included, but the solution is sought with metaheuristic methods and past data. Stage II-B is the stage where the Markov process, which is the distinctive feature of this study, is included. Table 12 is a comparison table of these stages. In the last column of the table, it is clearly seen how much FFVTD increases when the Markov process is included.

Table 12 shows the difference between the FFVTDs after the inclusion of the Markov process, that is, the amount of improvement in FFVTD. Table 12 is created as follows: For example, at TN = 1, the best FFVTD selected for past data is 5.4730e + 06 and the best FFVTD selected with the Markov process is 5.4731e + 06. Here, the amount of improvement brought to FFVTD by the inclusion of the Markov process is 1.0000E + 02. This process is repeated for all TNs and recorded in Table 12. As seen in Table 12, the limits computed in Stage II-B gave better results than the limits computed in Stage II-A.

Fig 12 shows the graph of the results given in Table 12. The differences between the fitness function values of the 4 different demand types belonging to Stage II-A and Stage II-B are shown as extra details in the graphs. The red lines represent Stage II-A and the blue lines represent Stage II-B. As can be seen in the details in the graph, the blue lines are above the red lines. This means that the results belonging to Stage II-B are better than Stage II-A. In other words, the approach involving the Markov process showed better results.

This obvious contribution is at a level that will affect all stages of the process directly or indirectly, starting with inventory cost and lost sales cost and continuing until customer satisfaction. This comparison clearly reveals the contribution of Markov process to inventory level optimization.

The proposed approach answered the main question and proved that the Markov process is effective in intermittent demand and inventory management. The findings obtained from the analysis of the Markov process clearly answered the main question. Thus, the effectiveness of the proposed approach is clearly proven as seen in Table 12 and Fig 12. The findings of the Markov process are in line with theoretical expectations and offer a new contribution.

Despite all these positive results, the proposed approach has some limitations. The biggest limitation of the study is the intermittent demand. It is mentioned in the Fitness Function and Test Data section that intermittent demand has certain statistics (ADI > 1.32 and ${CV}^{2}$<0.49). Although this situation limits the study in terms of intermittent demand, the proposed approach has the flexibility to work for all demand structures. Another limitation is that it is dependent on past period demands. While approaches such as Wagner-Whitin and Silver Meal methods work with future demands, the approach proposed in this study makes inferences about the future based on past period demands. Therefore, it is necessary to ensure that the data used with this approach is complete, accurate and consistent. Because intermittent demand is a demand that occurs within a certain time period. The time period considered affects the status of intermittent demand.

As seen in recent studies in the supply chain field, Robust Stochastic Optimization (RSO) and Conditional Value at Risk (CVaR) methods are very important approaches for uncertainty and risk management in decision-making processes [51–53]. Based on these studies, it is seen that in future studies, the fight against the uncertainty of intermittent demand will be further strengthened with uncertainty management and risk measurement methods such as RSO and CVaR. For difficult and large-scale problems such as intermittent demand problems, problem-specific heuristic algorithms [54], hybrid heuristics and metaheuristics, adaptive algorithms, self-adaptive algorithms, island algorithms, polyploid algorithms, hyperheuristics, and many other solution approaches with applications in different areas could be tried. Comparison of these approaches in terms of performance, solution time, ease of use by decision makers, compatibility with intermittent demand and inventory problems is important for future studies in this field.

### Managerial insights

This study combines Markov and metaheuristic approaches to combat the negative effects of intermittent demand. In this way, it is aimed to reduce the negative effects of intermittent demand originating from uncertainty. It is clearly seen that traditional methods are inadequate in combating intermittent demand and the positive effects of the proposed approach.

With this study, an upper limit for stock in the inventory management of intermittent demands has been found. However, in practical terms, determining only the upper limit may be insufficient. Because it is necessary to determine a reorder/production point for placing an order or for production. This also creates a lower limit. These two limits must be computed in an integrated manner. With this study, both the reorder/production point has been determined and the order/production quantity has been planned up to the upper limit. Optimizing these two limits in a balanced manner has provided the following major contributions from a managerial perspective:

1. Increasing customer satisfaction by eliminating lost sales,
2. Updating production plans according to optimized stock limits,
3. Reducing stock quantities with an upper stock limit,
4. Adjusting order quantities according to the variable structure of intermittent demand

This approach strengthens companies in terms of competitive advantage, not only in the short term but also in the long term, with its contributions to the supply chain. The fact that the proposed model has a flexible structure that can be used by every company, large or small, is also a great advantage for businesses.

## Conclusion

This research has integrated the Markov process with metaheuristic methods to provide stock level optimization of intermittent demands. Markov process traditionally focuses on demand forecasting in intermittent demand studies. In this study, the Markov process is at the focal point of a proactive stock level optimization. The Markov process and metaheuristic methods find stock limits to meet demand without estimating demand in an integrated manner. With these positive results, the proposed model analyzes the effects of the Markov process in intermittent demand stock management with metaheuristic methods and eliminates this deficiency in the literature.

The difficulty of estimating intermittent demand is a major problem for companies. Developing inventory models for products with intermittent demand is also difficult because of the demand structure. High holding costs or lost sales may occur if an appropriate inventory policy cannot be determined. To solve this problem, the proposed mathematical model is a good approach to both reduce holding cost and avoid lost sales. However, a feasible solution could not be found with the mathematical model. Therefore, a solution was sought in TSA and SA with the fitness function proposed in this study. However, calculating stock limits only for past demand does not fully account for the uncertainty of demand. The goal is to prevent the proposed model from memorizing the relevant process when it uses the past period demand. Approaching the process with the Markov chain led to better results when new demands are created.

Although the applicability of the model is based on intermittent demands, the model has the flexibility to work for all types of demands. The proposed model can be applied completely in real life problems. However, in this study, the model focuses on the assumption that the demand is intermittent. In this case, some limitations of the model emerge:

1 The data must provide statistics of intermittent demand (ADI and CV).
2 Data from past periods are needed.
3 Past data must be complete, accurate and consistent. Because intermittent demand occurs over a certain period of time.
4 Since a feasible solution cannot be provided by a mathematical model, metaheuristic methods were needed.

The most important key takeaway of the study is that the negative effects of the difficulty of predicting intermittent demands are reduced by optimizing stock levels with the Markov process. The Markov process clearly provides great benefits in intermittent demand stock level optimization. Another key takeaway is that the Markov process shows positive results in an integrated manner with metaheuristic methods.

The most important practical implication of this study is to ensure customer satisfaction in practice with stock level optimization. Appropriate stock levels prevent lost sales and reduce lost sales costs. The proposed approach makes great contributions to the stock management of businesses working with intermittent demands in practice. By establishing a balance between stock costs and lost sales costs, businesses update their production plans accordingly. Thus, excessive stock holding or lost sales situations are not encountered. Practical implications of the study can be listed as follows:

1. With the help of metaheuristic methods, feasible solutions for stock limits were provided faster and an integrated structure was designed into the Markov process and stock level optimization was provided for intermittent demands.
2. According to this study, stock limits determined with past data for intermittent demands give good results for past data. However, these limits did not give good results when trying to meet the negative effects of future uncertainty.
3. Uncertainty about future intermittent demand makes it difficult to evaluate the reliability of computed stock limits. This need was met with metaheuristic methods integrated with Markov chain. Stock limits of intermittent demand created with this method gave better results than stock limits computed using past data.
4. High costs (stock cost, lost sales cost, order cost, etc.) arising from intermittent demand in inventory management have decreased with the proposed Markov approach as seen in Table 12.

In future studies, the proposed approach can be expanded by making comparative analyses with hybrid heuristics, metaheuristics, adaptive algorithms, self-adaptive algorithms, island algorithms, polyploid algorithms, hyperheuristics. Another suggestion is to include methods such as fuzzy, RSO, data-driven and CVar in the model for uncertainty and risk in intermittent demand.
